# Assessing the threat of lone-actor terrorism: the reliability and validity of the TRAP-18

**DOI:** 10.1007/s11757-020-00596-y

**Published:** 2020-03-31

**Authors:** Angela Guldimann, J. Reid Meloy

**Affiliations:** 1 Fachstelle Forensic Assessment & Risk Management (FFA), Klinik für Forensische Psychiatrie, Psychiatrische Universitätsklinik Zürich, Lenggstr. 31, Postfach 363, 8032 Zürich, Switzerland; 2 Department of Psychiatry, University of California, San Diego, CA USA

**Keywords:** Structured professional judgment, Warning behaviors, Terrorism, Lone actor, Identification, Threat management, Strukturierte professionelle Risikobeurteilung, Warnverhalten, Terrorismus, Einzeltäter, Identifizierung, Bedrohungsmanagement

## Abstract

Terrorism, especially lone-actor terrorism, is considered a major national security threat in both North America and Europe. The threat of terrorism has many faces and violence can arise from all ideological extremes. The authors present the theoretical model and current empirical validation of the Terrorist Radicalization Assessment Protocol (TRAP-18), a structured professional judgment instrument for those engaged in risk assessment of persons of concern for acts of terrorist violence. It can be used independently of a particular ideology. The TRAP-18 consists of 8 proximal warning behaviors and 10 distal characteristics, and has been designed to help prioritize the imminency of risk in specific cases, and therefore determine the intensity of monitoring and active management a case requires. Research has demonstrated excellent interrater reliability, and promising content, criterion, discriminant, and predictive validity. More research is in progress. The TRAP-18 is currently used by counterterrorism experts in North America and Europe. It offers a useful approach for professionals who may be assessing and treating individuals of national security concern.

## Abstract (de)

Terrorismus, insbesondere durch sog. Einzeltäter, gilt sowohl in Nordamerika als auch in Europa als wesentliche Bedrohung der nationalen Sicherheit. Die Bedrohung durch Terrorismus hat viele Gesichter, und Gewalt kann aus allen extremen Ideologien entstehen. Die Autoren stellen in diesem Beitrag den theoretischen Hintergrund und die aktuellen empirischen Erkenntnisse zum Terrorist Radicalization Assessment Protocol (TRAP-18) vor. Der TRAP-18 folgt dem Ansatz der strukturierten, professionellen Risikobeurteilung und ist zur Einschätzung von Personen geeignet, die für mögliche terroristische Gewaltakte infrage kommen. Dabei kann das Instrument unabhängig von einer bestimmten Ideologie angewendet werden. Der TRAP-18 besteht aus 8 proximalen und 10 distalen Faktoren. Er wurde entwickelt, um den Anwender in konkreten Fällen bei der Priorisierung eines sich abzeichnenden Risikos zu unterstützen und dadurch die Intensität des Monitorings und aktiven Managements in einem Fall festzulegen. Forschungsergebnisse weisen auf eine exzellente Interrater-Reliabilität sowie auf vielversprechende Inhalts‑, Kriteriums- und Diskriminanzvalidität sowie prädiktive Validität hin. Weitere Studien sind aktuell in Arbeit. Derzeit nutzen Terrorismusexperten in Nordamerika und Europa den TRAP-18. Er kann als hilfreiche Herangehensweise dienen für Fachpersonen, die Personen bezüglich der Gefährdung der nationalen Sicherheit einschätzen bzw. behandeln müssen.

## Introduction

*And I call for my Muslim brothers and sisters all over the world to take part in jihad, and to fight for the dominance of this religion, as much as each of you can. If you can’t join your brothers on the front lines, then fight for Islam in your countries. And if you live in Europe, then fight against those pigs, each to his own abilities. May Allah grant us success in [this fight]. I pledge myself to Allah and vow to shed as much blood as it needs for Islam to prevail. I pray for Allah to pave the way for me to kill those infidels who fight Islam and Muslims *(Translation from YouTube video in Arab language, since removed).

A.A., then 24 years old from Tunisia, filmed himself and pledged this vow a few weeks before he committed an act of terror while driving a truck into a Christmas market near Berlin Memorial Church on December 19, 2016. Twelve people died and more than 50 were severely injured. Böckler et al. (2017) analyzed this complex case through the lens of the *warning behaviors* which are indicators of accelerating risk for targeted violence (Meloy et al. 2012). These warning behaviors are part of the Terrorist Radicalization Assessment Protocol (TRAP-18), a structured professional judgment instrument (SPJ) that will be introduced in this contribution as a way to help professionals prioritize cases of targeted terrorist violence concerning their imminency of risk—regardless of the terrorists’ particular ideology (Meloy 2017). 

## Challenges of terrorism

Risk assessment and prevention of terrorist attacks are daunting and challenging tasks. The challenge starts with the insight that a legally or scientifically accepted definition of terrorism that serves as a gold standard is missing (Leygraf 2014). But ideologies which drive such acts of violence toward noncombatant civilians share two common characteristics (Meloy 2017). First, they are acts of targeted violence, that is, intended and purposeful events which are virtually always the culmination of a *pathway toward violence*. These actions are not impulsive and typically not a reaction to an imminent threat, which define most violence among individuals (Meloy 2006). Second, as Nedopil (2014) has noted, not only is a target selected but also an audience. The terrorist hopes that society will respond with insecurity, fear and a growing mistrust toward each other. Bakunin, the 19th century anarchist, called it “propaganda of the deed” (Meloy 2017).

Terrorism, especially lone-actor terrorism, is considered a major national security threat in both North America and Europe, while acts of terrorism are most frequent in the Middle East (Institute for Economics and Peace 2019). Lone actors do not operate as steady members of a large terrorist cell or organization. Nonetheless, lone actors are inspired and radicalized by writings and postings of like-minded individuals and charismatic leaders through virtual and face-to face contact. The exact line between “lone actors” and operating (small) cells are blurred in some cases. A.A. seemed to have had ties and support from IS (Bundesministerium für Justiz und Verbraucherschutz 2017). In contrast the 21-year-old attacker from Kosovo who killed two US soldiers at Frankfurt airport seemed to have radicalized himself to a great extent through the Internet (Böckler et al. 2016). Until recently the media and law enforcement agencies have focused on violent Islamic jihadists (e.g., the attack on the journalists for Charlie Hebdo in France in 2015), but serious threats from right-wing ideologies and specific groups (Reichsbürger, etc.) are not to be underestimated—and are growing— (Bundesministerium des Innern, für Bau und Heimat 2019), since threats and violence can arise from all extremes (e.g., attack on a mosque in Christchurch, New Zealand, 2019). The violent method often overshadows and becomes the ideology.

Leygraf (2014) forensic psychiatrically evaluated one end of the extreme. He gave expert reports to the courts concerning 29 Islamistic terrorists between 2000 and 2013. In the “immigrant” group (*n* = 19), no psychopathological traits were detected. The author described them as primarily dissocial offenders, as well as offenders who had failed to cope with their way of life or their personal aims in life. The other ten offenders grew up in Germany. Three subjects suffered from a schizophrenic psychosis and two had a primarily dissocial personality. The remaining subjects showed some special features in their developmental background (identity crises), but without any noticeable uniform background pattern that pointed toward Islamic terrorism. No single distinctive profile of “the Islamistic terrorist” emerged.

But do lone actors with varying ideologies differ significantly on personal, psychopathological and social indicators or violence risk factors? Bouhana et al. (2018) compared right-wing lone actors with other lone actors (e.g., jihadist). They found some differences: Right-wing lone actors committed significantly more violent offenses in the past and used firearms more often than bladed instruments during the attack. But the authors concluded that right-wing lone actors and the individuals adhering to other ideologies showed far more similarities than differences on the majority of indicators, including all indicators related to motivation, capability, as well as pre-attack warning signs. Bouhana et al. (2018) addressed the extent to which risk assessment tools and processes should be tailored to a specific ideology. They concluded that some specificity and variability in indicators are likely and possible due to social and self-selection effects, but that it can be assumed that underlying processes and mechanisms are similar among all lone-actor terrorists (Gill 2015). More research is needed, of course, but the state of the science suggests that terrorists from a particular ideology do not show a singular profile as a result of their group affiliation, nor can they be effortlessly distinguished from individuals with other ideological backgrounds in terms of psychopathology, personality or other specific risk factors.

Complicating the challenging matter are the very low base rates for such terrorist acts, which preclude accurate prediction. According to Borum (2015) there are four key guidelines when developing a terrorism-related threat assessment scheme: (a) broadly conceiving risk factors from both an idiographic and nomothetic perspective; (b) being open to different pathways and different roles in terrorism (e.g., leader or supporter) for a person of concern (POC); (c) recognizing that large group factors (such as age) may or may not apply to the POC and (d) emphasizing prevention/management of risk rather than prediction.

It is also very important that we distinguish between prediction and prevention, whether in threat assessment or public health in general. The current pandemic of the corona virus, for example, is being managed through the treatment of symptomatic individuals, but also the identification of *risk factors* and their management in the most vulnerable individuals in society—without trying to predict which of those most vulnerable individuals will become symptomatic. Prevention is attainable if the focus is upon fact-based behaviors of concern, and threat management is employed to mitigate such behaviors (Meloy et al. 2014). Threat assessment and violence risk assessment are similar but do also emphasize different aspects. Threat assessment and management focuses upon the identification, assessment, and management of a POC in real time—with close attention paid to the target and the situation— while the traditional task of “violence risk assessment” is the determination of relative risk in an individual at a particular point in time by determining the base rate of violence for the group within which he or she belongs. The latter tends to be a more static task, while the former is often more dynamic and urgent.

## TRAP-18: warning behaviors and distal characteristics

Proximal behaviors of concern were included in the warning behaviors, first introduced through a sample of German public figure attackers (Hoffmann et al. 2011). The concept of “warning behaviors” originated in the studies of the Fixated Research Group concerning abnormal communications and approaches to the British Royal Family (James et al. 2007) and were termed by other authors as “pre-attack signals” (Dietz and Martell 1989). Since a structured approach and typology of such warning behaviors was missing in the threat assessment literature, they were organized and operationalized after intensive research on targeted or intended violence, discussions with colleagues, and the casework experience of the original authors. The 8 warning behaviors capture behavioral or psychological patterns which constitute change and may evidence accelerating risk (Meloy et al. 2012). They contain dynamic rather than static factors, since the former typically offer more substantial contributions to the assessment and management of short-term violence which is usually the focus of threat assessment (Douglas and Skeem 2005). The warning behaviors are not discrete variables, but patterns for analysis (Guldimann et al. 2013). They have been researched in various samples of intended violence such as school shooters vs. school threateners, intimate partner homicide (Meloy et al. 2014), mass murders in Germany (Allwinn et al. 2019) and Switzerland (Ilic and Frei 2019), and some of the warning behaviors have been included in the PRAT, a tool developed by the Swedish Defence Research Agency to discover linguistic markers in the written (online) communication of lone actors (Akrami et al. 2018).

Against this empirical background the 8 warning behaviors were included as proximal indicators of violence risk in the Terrorist Radicalization Assessment Protocol (TRAP-18). Second, 10 distal characteristics of the lone-actor terrorist were derived from studying the extant empirical and theoretical research on terrorism, and Meloy’s experience in directly and indirectly assessing both foreign and domestic lone-actor terrorists (Meloy and Yakeley 2014, Fig. 1). Guiding the development of the TRAP-18 were also Monahan’s (2012) recommendations that domains of (a) grievances, (b) ideologies, (c) affiliations and (d) moral emotions should be included in risk assessment instruments for terrorist violence.

**Fig. 1:**
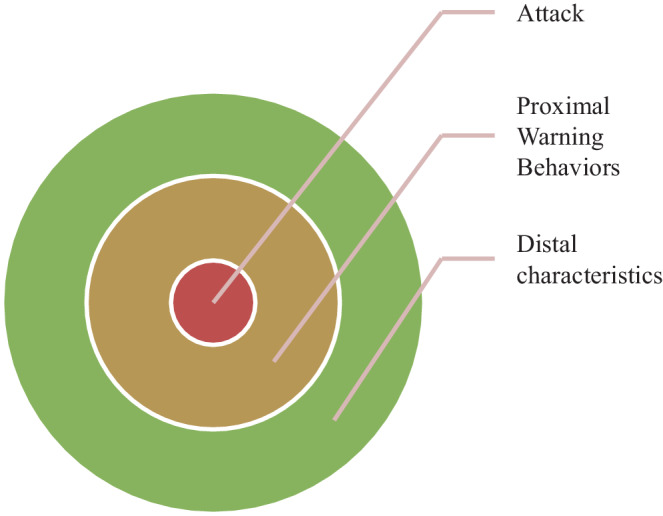
Suggested relationship between TRAP-18 indicators and attack

The 18 indicators are coded as either present or absent if there is sufficient evidence to make this determination. The TRAP-18 is not a psychological test, nor an actuarial risk assessment instrument. There are no empirically derived cutoffs for the TRAP-18 since it is a structured professional judgment instrument. Nevertheless, the model advances the hypothesis that one proximal warning behavior is necessary for active threat management, while the presence of only distal characteristics means the case should be monitored. The following TRAP-18 definitions are abbreviated and should not be used as the basis for threat assessment without training in the use of structured professional instruments and the TRAP-18 (Meloy 2017; gifrinc.com, Table 1).

**Table 1:** TRAP-18: 8 proximal and 10 distal indicators

Proximal Warning Behaviors	Distal Characteristics
Pathway	Personal Grievance and Moral Outrage
Fixation	Framed by an Ideology
Identification	Failure to Affiliate with an Extremist or Other Group
Novel Aggression	Dependence on the Virtual Community
Energy Burst	Thwarting of Occupational Goals
Leakage	Changes in Thinking and Emotion
Last Resort	Failure of Sexually Intimate Pair Bonding
Directly Communicated Threat	Mental Disorder
–	Creativity and Innovation
–	Criminal Violence

### The 8 proximal warning behaviors

1. *Pathway* warning behavior is research, planning, preparation, or implementation of an attack (Fein and Vossekuil 1999; Calhoun and Weston 2003).
2. *Fixation* warning behavior indicates an increasingly pathological preoccupation with a person or a cause, accompanied by a deterioration in social and occupational functioning (Mullen et al. 2009).
3. *Identification* warning behavior indicates a psychological desire to be a pseudocommando (Dietz 1986; Knoll 2010), have a warrior mentality (Hempel et al. 1999), closely associate with weapons or other military or law enforcement paraphernalia, identify with previous attackers or assassins, or identify oneself as an agent to advance a particular cause or belief system (Meloy et al. 2012).
4. *Novel Aggression* warning behavior is an act of violence that appears unrelated to any targeted violence pathway and is committed for the first time. It is often a test of one’s capability to carry out an act of violence (Meloy et al. 2012).
5. *Energy Burst* warning behavior is an increase in the frequency or variety of any noted activities related to the target, even if the activities themselves are relatively innocuous, usually in the hours, days or weeks before the attack (Odgers et al. 2009; Meloy et al. 2012).
6. *Leakage* warning behavior is the communication to a third party of an intent to do harm to a target through an attack (Meloy and O’Toole 2011).
7. *Last Resort *warning behavior is evidence of a “violent action imperative” and “time imperative” (Mohandie and Duffy 1999); it is often a signal of desperation or distress.
8. *Directly Communicated Threat* warning behavior is the communication of a direct threat to the target or law enforcement beforehand (Meloy et al. 2012).

### The 10 distal characteristics of the TRAP-18

1. *Personal Grievance and Moral Outrage* joins both personal life experience and particular historical, religious, or political events. The personal grievance is often defined by a major loss in love or work, feelings of anger and humiliation, and the blaming of others. Moral outrage is typically a vicarious identification with a group that has suffered, even though the POC has usually not experienced the same suffering, if any at all.
2. *Framed by an Ideology* is the presence of a belief system that justifies the POC’s intent to act. It can be a religious belief system, a political philosophy, a secular commitment, a one-issue conflict, or an idiosyncratic justification (Simon 2013; Meloy and Yakeley 2014).
3. *Failure to Affiliate with an Extremist or Other Group* is defined by the actual failure or rejection of the POC from a group that he wants to join.
4. *Dependence on the Virtual Community* is evidence of the POC’s active communication with others through social media, chat rooms, e‑mails, listservs, texting, tweeting, and so forth about his radical or extreme beliefs.
5. *Thwarting of Occupational Goals* is a major setback or failure in a planned occupational life course.
6. *Changes in Thinking and Emotion* is indicated when thoughts and their expression become more strident, simplistic, and absolute. Argument ceases, and preaching begins. Persuasion yields to imposition of one’s beliefs on others. There is no critical analysis of theory or opinion, and the mantra, “Do not think, just believe” is adopted. Emotions typically move from anger and argument, to contempt and disdain for others’ beliefs, to disgust for the outgroup and a willingness to homicidally aggress against them. Violence is cloaked in self-righteousness and the pretense of superior belief. Humor is lost.
7. *Failure of Sexually Intimate Pair Bonding* is coded if the POC has historically failed to form a lasting sexually intimate relationship.
8. *Mental Disorder* is coded if there was evidence of a major mental disorder by history or at present.
9. *Greater Creativity and Innovation* is coded if there is evidence of tactical thinking “outside the box;” The act being planned has not been done before in contemporaneous times, or is likely to be imitated by future offenders (Simon 2013; Meloy and Yakeley 2014).
10. *Criminal Violence* is coded if there was evidence of instrumental criminal violence by history prior to the act of terrorism (e.g., history of armed robberies or planned assaults).

## Current state of the science on the TRAP-18

The TRAP-18 needs to stand the test of empirical validation. The empirical findings seem encouraging, but more extensive and independent research is needed since there is a clear authorship bias when the developers do research with their own instrument (Singh et al. 2013).

Meloy et al. (2015a) analyzed 19 cases and 22 individuals who carried out acts of terrorism in Europe between 1980 and 2015. Seven of them formed autonomous cells (two or more offenders whose actions were not commanded nor controlled by a terrorist organization, e.g., German National Socialist Underground). Only two of the attacks were not lethal. The mean interrater reliability (Cohen’s kappa) was 0.895 and ranged from 0.69–1.0 for the warning behaviors and 0.75–1.0 for the distal characteristics (good to excellent across all variables). This study lacked a non-terrorist control group. The results can only be interpreted as a measure of the goodness of fit with real world terrorists. As a form of content validity it can be reported that the majority of lone actors were positive on 13 of 18 TRAP-18 variables (72%); Individuals acting in autonomous cells were likewise positive for 13 of 18 variables (72%) of the TRAP-18. Both groups showed a frequency of >70% on the distal factors of *personal grievance and moral outrage, framed by an ideology, thwarting of occupational goals, and changes in thinking and emotion.* There was no significant difference between any of the TRAP-18 indicators when the terrorists who acted alone (*n* = 15) were compared with those in autonomous cells (*n* = 7), except for the significant finding that the latter had a more frequent history of criminal violence (100%).

Meloy and Gill (2016) used an open source sample of 111 lone-actor terrorists (1990–2014) from the U.S. and Europe to further validate the TRAP-18. Terrorism was defined as “the use or threat of action where the use or threat is designed to influence the government or to intimidate the public or a section of the public, and/or the use or threat is made for the purpose of advancing a political, religious, or ideological cause” (Gill et al. 2013, p. 2). Seventy per cent of the terrorists were positive for at least half or more of the TRAP-18 indicators. Seventy-seven per cent or more evidenced four proximal warning behaviors: *pathway, fixation, identification, and leakage*, consistent with other domains for targeted violence (Meloy et al. 2014).

When the sample was divided into Islamic terrorists (*n* = 38), extreme right-wing terrorists (*n* = 43), and single-issue terrorists, mostly anti-abortionists (*n* = 30), there were no significant differences across the 18 indicators except for four: Islamic extremist lone actors were significantly more likely to display *dependence on the virtual community* than the single-issue terrorists. Extreme right-wing lone actors were significantly less likely to display *personal grievance and moral outrage, thwarting of occupational goals, and fixation warning behaviors* than either the Islamic extremists or the single-issue terrorists. Single-issue lone actors were significantly less likely to display *dependence on virtual communities *than the Islamic extremists. More research is warranted, but the results are in line with Bouhana et al. (2018) findings that a clear profile dependent upon ideology is not to be expected.

Finally, successfully executed (*n* = 67) and planned but thwarted (*n* = 44) attacks were compared. A thwarted attack covered plots that were developed by lone-actor terrorists that were interrupted, uncovered, or stopped by law enforcement and subsequently led to a conviction. The successful attackers differed on five TRAP-18 indicators from the thwarted group. They were significantly more *fixated, creative and innovative, and failed to have a sexually intimate pair bond*; and were significantly less likely to have displayed *pathway warning behavior *that was discovered beforehand and be *dependent on a virtual community* of likeminded individuals. Effect sizes were small to medium (Φ = 0.19–0.32). Detectable *pathway behavior* was evident in 72% of the successful and in 93% of the thwarted group. Whether this last difference was based on luck, stealth, or inadequate intelligence gathering is not clear. The significantly more prevalent *fixation warning behavior* in the successful attackers describes an intensity of pursuit and preoccupation with the subject, often accompanied by social deterioration in love and work and the tendency to socially withdraw. A history of *failed sexual pairbonding* in general—like in the group of successful attackers—also lowers the risk of an intimate becoming familiar with one’s activities and disrupting the operation. On the other hand, a failure to establish long lasting relationships could intensify *fixation* on a subject or a person as a substitute for real relationships. Less *dependence on the virtual community *means a lessened chance of having one’s postings or social media communication picked up by a third party or discovered by authorities. *Creativity and innovation*, another distal characteristic more frequent among the successful attackers, contributes to the success of the attack. From an operational point of view, these findings make sense but need to be replicated in further studies.

In an independent study by Challacombe and Lucas (2018), 58 US domestic terrorists (Sovereign Citizens: typically extreme right wing anti-government)—30 who had committed violent or dangerous actions and 28 who committed nonviolent criminal actions in a 10-year frame (2004–2014)—were compared. Interrater reliability was excellent (kappa = 0.76). These TRAP-18 indicators significantly correlated with violence with medium to large effect sizes (Φ = 0.33–0.70): the proximal warning behaviors of *pathway, identification, leakage, and last resort*. The distal characteristics of *personal grievance and moral outrage, framed by ideology, thwarting of occupational goals, and criminal violence* were all more frequent among the violent right-wing extremists. A binary logistic regression using the summed TRAP-18 score was performed and correctly explained 44–59% of the variance. The TRAP-18 total coding correctly classified 75.9% of the cases as either violent or nonviolent (odds ratio was 2.10; *p* = 0.000).

Another comparative study of North American terrorist attackers (*n* = 33) and persons of national security concern who were successfully risk managed (*n* = 23) found significant differences with medium to large effect sizes (Φ = 0.35–0.70) across half of the TRAP-18 indicators (Meloy et al. 2019). The subjects in the study were an ideological mix of violent jihadists, extreme right-wing nationalists, and single-issue terrorists. The proximal warning behaviors which differentiated the attackers from those subjects of concern were *pathway, identification, energy burst, last resort, and the absence of a directly communicated threat.* The distal characteristics which were more frequent in the attackers were* ideological framing, changes in thinking and emotion, and creativity and innovation.* A follow-up study using the Meloy et al. (2019) sample and multidimensional scaling analysis—rarely done in terrorism research—demonstrated that the proximal warning behaviors clustered together among the attackers, did not cluster among the non-attackers, and showed less of a clustering and association of distal characteristics. Distal characteristics between attackers and non-attackers were not significantly different. These findings further support the theory of the TRAP-18 by visually showing the co-occurrence of the proximal warning behaviors in the attackers, but not in the non-attackers despite concern for their behaviors (Goodwill and Meloy 2019).

What has emerged across all the targeted violence research to date is the ubiquity of the *proximal warning behaviors of pathway, fixation, identification, leakage, energy burst, and last resort*. Within the lone-actor terrorist domain, the evolution from *fixation to identification*—what one thinks about all the time (preoccupation), to what one becomes (self identity)—may be a critical marker for mobilization for violence (Challacombe and Lucas 2018; Meloy et al. 2019). This transition should be closely monitored by the threat manager in face-to face discussions, written statements in the real or virtual world, or other symbolic actions initiated by the POC.

A *directly communicated threat* did not emerge in the existing studies as a critical warning behavior in lone-actor terrorists. This finding is in line with other targeted violence research (except for intimate partner homicide) such as attacks on public figures or school shootings and makes sense from an operational perspective (Meloy et al. 2014). During the writing of this paper the attack in Hanau, Germany, occurred. The attacker killed 9 people with immigration backgrounds, and finally his mother and himself. The investigation is still ongoing and final conclusions are not warranted, but at the point of this writing there is no evidence of a *directly communicated threat* beforehand. To be clear, a *directly communicated threat* should always be seriously assessed; but the lack of such a threat in any case should not be mistaken as an “all clear sign.”

Monahan and Steadman (1996) first introduced a weather analogy to violence risk assessment which appears in the context of the TRAP-18 to be a useful metaphor: The presence of a cluster of TRAP-18 distal characteristics, along with the *absence* of all proximal warning behaviors, indicate there may be storm clouds on the horizon. It is still uncertain if a storm will form, but a “Watch” needs to be initiated. In the world of threat assessment, this means that the case should be monitored and reviewed on a regular basis. Monitoring should be done contemporaneously in both virtual and terrestrial worlds. The case does not yet warrant more commitment of active management resources. The presence of *any one* proximal warning behavior, on the other hand, means the storm may be in one’s backyard. A “Warning” needs to be initiated. In the world of threat assessment, this means active management: face-to-face interview with the POC and/or collateral interviews with family or peers; review of records (e.g., military, criminal, residence, police incidents, employment); social media monitoring; civil commitment, release, and discharge planning; safety plan development for school, work, home and the community at large; and obtaining signed consents to communicate with the POC’s mental health professional to monitor progress (Goodwill and Meloy 2019).

As practitioners, we are the first to acknowledge that any given case is far more complex than empirical results or analogies might suggest. For example, the clustering of three “strong” distal characteristics with the proximal warning behaviors in the Goodwill and Meloy (2019) most recent study—namely, *personal grievance and moral outrage, ideological framing, and changes in thinking and emotion*—complicate the weather analogy. A storm is forming, but its arrival may not be imminent. However, active management of the case is likely to be warranted if these three strong distal characteristics are present—even in the absence of known proximal warning behaviors.

## Conclusions and further directions

The TRAP-18 has been subjected to peer-reviewed studies testing its reliability and validity, including research independent of its developer (Challacombe and Lucas 2018; Garcia-Andrade et al. 2019). The retrospective nature of the studies and the small sample sizes have to be taken into account when interpreting the results. Further research is warranted and potential weaknesses of the TRAP-18 (e.g., lack of protective factors) need to be taken into account, but the results so far seem promising. There are other instruments used to assess risk of terrorist violence, including the ERG 22+ (Extremism Risk Guidelines; Lloyd and Dean 2015) and the VERA (Violent Extremism Risk Assessment; Pressman 2009). Multimethod approaches are best for the assessment of terrorism risk, and other instruments that correlate with violence risk, such as the HCR-20 V3 (Historical-Clinical-Risk; Douglas et al. 2013) and the PCL‑R (Hare 2003) are also recommended.

The application of instruments is only the first step. Threat management can only succeed if an interdisciplinary approach is guaranteed. Police, law enforcement, forensic and general mental health professionals and other experts must join forces. We have not elaborated on the issue of the prevalence of mental disorders in terrorists, but while some violence may stem from long held racist attitudes—such as is evident in the globalization of the white supremacist movement— other violence is the result of a combination of mental illness and radicalization in the attacker. The potential for human loss is the same, but management strategies to prevent such attacks are quite different (e.g., ensuring medical–psychiatric treatment vs. no such treatment). The case must be analyzed on an individual level since a “one size fits them all” strategy is likely doomed to fail (Habermeyer and Guldimann 2019). The paradox is that the professional involved will usually never know whether the (terrorist) act would have occurred if he or she had not intervened. Nevertheless, the operational necessity to focus upon fact-based behaviors in the present, rather than static diagnoses or historical factors in the investigation of POCs, is the key to successful prevention (Meloy et al. 2015b).

Further research should also focus on disengagement factors in terrorists, and the potential of linguistic analysis should be given more weight, since the “terrorist in the making” is drawn to the cyberprints of like-minded spirits and leaves his own imprint in the digital world. According to Gill (2015), reinforcement of beliefs, seeking legitimization for their actions, disseminating propaganda, recruitment, attack preparations, overcoming obstacles and signaling the attack are motives to get and stay connected in virtual reality.
